# Trauma-Induced Thyroid Storm After Road Traffic Accident Polytrauma

**DOI:** 10.7759/cureus.107643

**Published:** 2026-04-24

**Authors:** Alya Elmaimona H Abdelmageed, Malaz S Younis, Sahar Alomar, Ahmed Hamad, Aymen S Mohammed, Zisis Touloumis

**Affiliations:** 1 Emergency Department, Omdurman Military Hospital, Khartoum, SDN; 2 Surgery Department, King Saud Medical City, Riyadh, SAU; 3 Plastic Surgery Department, Dr. Mohammad Alfagih Hospital, Riyadh, SAU; 4 Trauma Center, King Saud Medical City, Riyadh, SAU; 5 General Surgery Department, Dr. Sulaiman Al Habib Medical Group, Riyadh, SAU; 6 General Surgery Department, Dr. Mohammad Alfagih Hospital, Riyadh, SAU; 7 Trauma Surgery Department, King Saud Medical City, Riyadh, SAU

**Keywords:** induce, road traffic accident, thyroid condition, thyroid storm, trauma

## Abstract

Thyroid storm (TS) is a rare, life-threatening exacerbation of hyperthyroidism, where the thyroid gland produces too much of the thyroid hormones, often triggered by causes such as infection or surgery. Although rare, trauma can precipitate the transition from thyrotoxicosis to TS. Though typically seen in patients with known hyperthyroidism - especially Graves' disease - the onset of TS can be unpredictable and rapidly fatal if not promptly addressed. We present a 20-year-old male who developed TS following a road traffic accident. On arrival, the patient was in hypovolemic shock and subsequently underwent an emergency laparotomy and splenectomy for a grade V splenic injury. Despite intensive care unit (ICU) support, the patient showed persistent tachycardia, high fever (40.8°C), and reduced Glasgow Coma Scale (GCS). Thyroid function tests (TFT) confirmed biochemical thyrotoxicosis. This case highlights trauma as an uncommon but significant precipitant of TS, especially in patients unaware of underlying thyroid disease. Diagnostic delays may occur as symptoms mimic common post-trauma complications. In trauma patients with unexplained systemic instability, TS should be considered since early recognition and targeted treatment are vital to reduce morbidity and mortality.

## Introduction

Thyroid storm (TS) is an uncommon but critical complication of hyperthyroidism, usually precipitated by stressors like infection or surgery. In some cases, traumatic injury may act as a catalyst, driving the progression from thyrotoxicosis to TS [1,2].

The occurrence of TS is in approximately 1-10% of hospitalized patients with thyrotoxicosis. It is more prevalent in females and individuals with Graves' disease [3]. Despite modern advancements and supportive measures in treating this condition, the mortality associated with TS is estimated to be 8-25% [4], underscoring its status as a potentially fatal endocrinological emergency.

TSs are often distinguished by pronounced clinical manifestations of severe thyrotoxicosis, usually presenting with a triad of hyperthermia, tachycardia, and altered mental status. It is often precipitated by factors such as infection, iodine-containing contrast agents, pregnancy, surgery, and certain medications like amiodarone [5]. Although less common, trauma is also recognized as a potential precipitating factor [6].

The conversion of thyrotoxicosis into TS typically requires a triggering event, and while trauma is a rarer trigger, it remains a recognized precipitant [1]. Due to the broad systemic effects of thyroid hormones, the symptoms of TS can be varied and often nonspecific [2], complicating its diagnosis, particularly in trauma patients without a known medical history. Symptoms such as altered consciousness and tachycardia may be erroneously attributed to the trauma itself [5].

Given these challenges, maintaining a high index of suspicion, coupled with a thorough patient history as well as awareness of TS risk factors, is essential for reducing the associated mortality and morbidity [5].

## Case presentation

A 20-year-old male was transferred to our hospital as a critical care patient following a motor vehicle accident that resulted in multiple traumatic injuries. Prior to transfer, he was stabilized and treated according to the advanced trauma life support (ATLS) protocol. He sustained polytrauma with a blunt abdominal injury, necessitating an exploratory laparotomy and splenectomy. Following intubation, the patient was placed on mechanical ventilation before being referred to our trauma team for further management.

Additionally, he suffered a fracture of the right forearm, which was initially managed with a backslap and later treated surgically with open reduction and internal fixation. During his hospital course, the patient developed several complications, including transfusion-related acute lung injury (TRALI), ventilator-associated pneumonia, and septic shock, from which he eventually recovered. He also sustained a diffuse axonal injury and experienced episodes of atrial fibrillation with rapid ventricular response.

Due to the preceding respiratory failure, he required reintubation and continued mechanical ventilation, along with treatment consisting of antibiotics, antiarrhythmics, and other supportive therapies. Upon admission to our ICU, the patient was intubated with an oxygen saturation of 97%. He initially presented in hypovolemic shock but was quickly stabilized hemodynamically. His Glasgow Coma Scale (GCS) score was initially 3/15 but improved following resuscitation.

**Figure 1:**
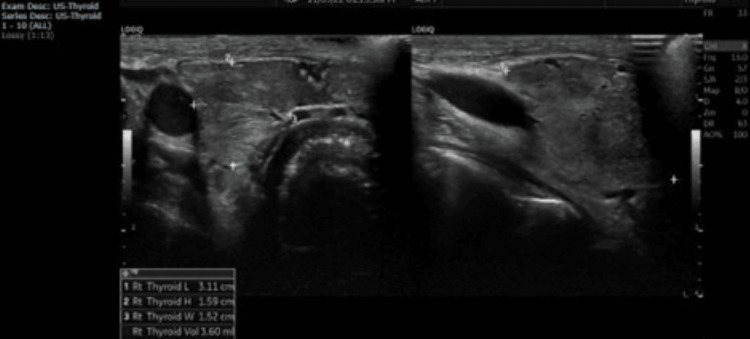
Normal size and shape of both thyroid lobes (each with an approximate volume of 3.6 mL)

**Figure 2:**
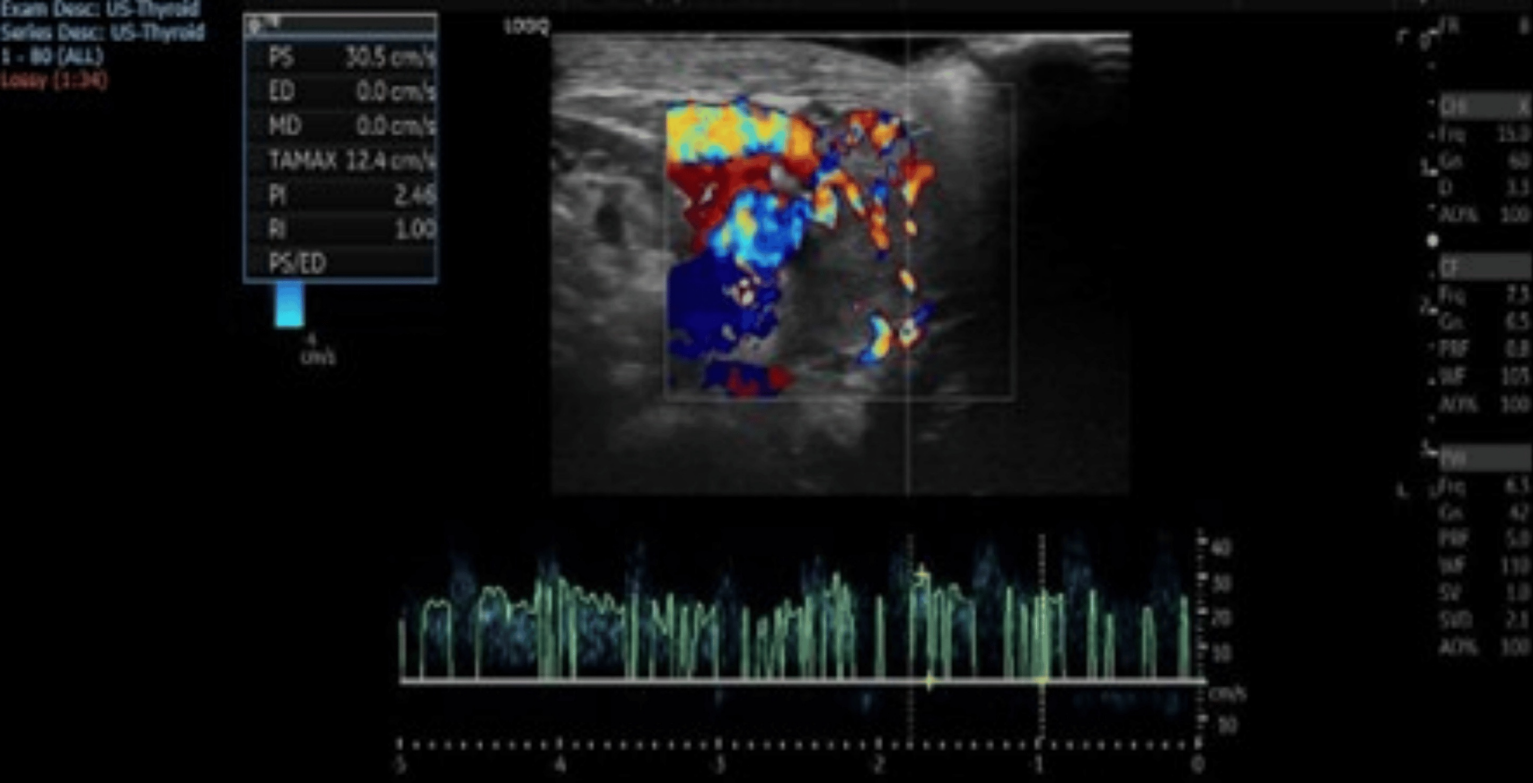
Homogeneous echo pattern and increased parenchymal vascularity, with peak systolic velocity (PSV) reaching about 43 cm/s

**Figure 3:**
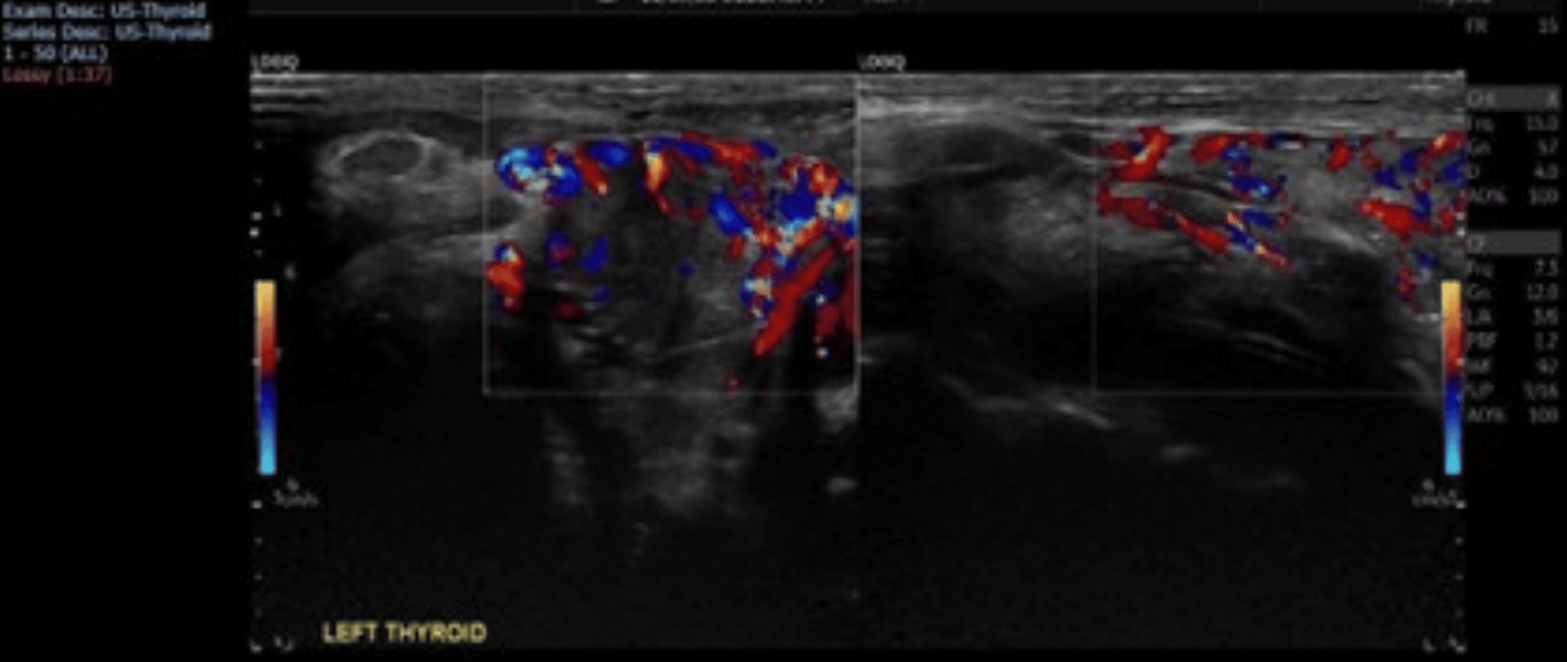
Homogeneous echo pattern and increased parenchymal vascularity, with peak systolic velocity (PSV) reaching about 43 cm/s

Despite close monitoring and standard trauma management, the patient continued to exhibit a persistently low GCS with persistent tachycardia and a high fever of 40.8°C. Further investigations revealed abnormal thyroid function tests seen in Table 1, raising suspicion of TS. Ultrasound examination of the thyroid gland showed no gross nodules, calcifications, or significantly enlarged lymph nodes. However, the findings were suggestive of thyrotoxicosis, with mild hypervascularization of the thyroid gland indicating possible compensatory changes, as seen in Figures 1-3.

**Table 1:** Laboratory results of our patient's thyroid function tests TSH: thyroid-stimulating hormone; T3: triiodothyronine; T4: thyroxine

Thyroid function tests	1st Value	2nd Value	Normal range	Conventional units
TSH	0.012 mIU/L	0.005 mIU/L	0.40-5.0 mIU/L	Same
T4	45.5 pmol/L	40.13 pmol/L	12-22 pmol/L	3.5-7.9 ng/dL
T3	9.25 pmol/L	9.51 pmol/L	3.1-6.8 pmol/L	1.98-4.42 pg/mL

The combination of severe clinical symptoms, abnormal laboratory results seen in Tables 2-3, and ultrasound findings all pointed to a progression from thyrotoxicosis to TS. The endocrinology team was promptly consulted, and the patient was treated with thionamide and beta-blockers to alleviate the acute effects of the storm. Subsequently, propylthiouracil and hydrocortisone infusions were administered to control the remaining symptoms and stabilize his condition. The patient remained in the ICU, followed up by the associated departments, until his condition improved. He was then transferred to the ward after two weeks and discharged a week later.

**Table 2:** Laboratory results of our patient's biochemistry lab tests PTH: parathyroid hormone

Biochemistry lab tests	Lab result	Normal range	Conventional units
Sodium	132 mmol/L	135-145 mmol/L	Same
Potassium	4.99 mmol/L	3.5-5.0 mmol/L	Same
PTH	10.33 ng/L	10-65 ng/L	10.33 pg/mL
Calcium	2.43 mmol/L	2.1-2.6 mmol/L	9.72 mg/dL
Vitamin D	50.04 nmol/L	>50 nmol/L	20 ng/mL
Urea	3.9 mmol/L	2.0-7 mmol/L	23.4 mg/dL
Creatinine	20 umol/L	55-120 umol/L	0.23 mg/dL

**Table 3:** Laboratory results of our patient's hematology lab tests INR: international normalized ratio; PT: prothrombin time; PTT: partial thromboplastin Time; WBC: white blood cell count Peripheral blood smear: normocytic normochromic anemia, absolute neutrophilia, left shift, activated lymphocytes

Haematology lab tests	Lab result	Normal range	Conventional units
Haemoglobin	7.6 g/dL	Men: 13.2-16.6 g/dL	Same
WBC	15.91 x 10^9^/L	4.0-11.0 x 10^9^/L	Same
Platelets	759 x 10^9^/L	150-400 x 10^9^/L	Same
INR	1.09	0.8-1.1	Same
PT	14.8 sec	10-14 sec	Same
PTT	45.5 sec	25-35 sec	Same

## Discussion

Hyperthyroid patients usually complain of symptoms such as heat intolerance, diaphoresis, palpitations, restlessness, and anxiety. During TS, these benign symptoms are usually exacerbated, becoming life-threatening [6]. Therefore, recognizing the varied symptomatology is critical for early diagnosis.

To aid this, clinical scoring systems, such as the Burch-Wartofsky Point Scale (BWPS) and the Japanese Thyroid Association (JTA) criteria, have been developed, providing structured approaches for diagnosing TS based on clinical features and laboratory findings. These tools help clinicians differentiate TS from less severe thyrotoxicosis and guide urgent management decisions [1].

Diagnosing TS is primarily clinical, given the significant variability in presentation. Patients often display multi-system involvement, including fever, cardiovascular abnormalities, and neuropsychiatric symptoms. Hyperpyrexia is a hallmark, with temperatures reported as high as 108.3°F (42.4°C) [1]. Central nervous system manifestations range from anxiety and confusion to severe delirium and psychosis [1]. Cardiovascular complications include sinus tachycardia, increased systolic blood pressure, atrial and ventricular tachyarrhythmias, ventricular dysfunction, and high-output congestive heart failure [2]. On examination, an enlarged thyroid gland with bruit or palpable thrill may be noted [4].

Progression from thyrotoxicosis to TS occurs in less than 10% of hospitalized patients, usually precipitated by infection, surgery, emotional stress, diabetic ketoacidosis, radiocontrast iodine exposure, or amiodarone therapy [7]. However, predicting this progression remains challenging. Trauma, though rare, has been recognized as a precipitating factor, accounting for 3.9% of TS cases in a recent nationwide Japanese survey [8].

The management of TS is complex and requires a multimodal approach, best delivered by endocrinologists and intensivists. Supportive care includes intravenous hydration, oxygen supplementation, vasopressor support in cases of circulatory collapse, electrolyte correction, arrhythmia management, and aggressive treatment of hyperthermia.

Medical therapy targets stabilization of the hyperthyroid state while addressing systemic decompensation and the underlying trigger. Treatment is initiated by blocking new hormone synthesis using thionamides such as propylthiouracil (PTU) or methimazole. PTU is preferred in TS due to its additional ability to inhibit peripheral conversion of T4 to the more active T3 [2]. Notably, PTU carries risks of hepatotoxicity, whereas methimazole is associated with fewer severe adverse effects, making it the preferred agent in less severe thyrotoxicosis or maintenance therapy [9].

Following thioamide administration, iodine solutions, such as a saturated solution of potassium iodide (SSKI), are given to exploit the Wolff-Chaikoff effect, which acutely inhibits thyroid hormone release and reduces gland vascularity. This step is critical but must follow thioamide therapy to prevent increased hormone synthesis.

Beta-blockers play a vital role in controlling the severe tachycardia and other cardiovascular manifestations of TS. Propranolol is frequently used due to its additional effect of inhibiting the peripheral conversion of T4 to T3. Corticosteroids, such as hydrocortisone, are administered both to reduce peripheral conversion and to treat relative adrenal insufficiency in severe illness.

Effective control of tachycardia is essential to prevent cardiovascular collapse, arrhythmia, and heart failure. Basic management of cardiovascular abnormalities includes careful monitoring and intervention to stabilize hemodynamics while the underlying thyroid crisis is addressed [2].

Recent data show that TS remains associated with significant in-hospital mortality, although advances in intensive care have improved survival rates. In the United States, between 2004 and 2013, mortality rates in hospitalized thyrotoxic patients were significantly higher in those with TS than without, underlining the need for prompt diagnosis and proper treatment [10].

Additionally, nationwide studies from Japan highlight the rarity of TS, estimating an annual incidence of only 0.20 per 100,000 population. Despite its low frequency, the potential for misdiagnosis is still high, reinforcing the value of standardized diagnostic tools such as those established by the JTA [9].

Finally, treatment protocols based on large-scale surveys emphasize the importance of a stepwise, protocol-driven approach to improve outcomes. Early combination therapy with antithyroid drugs, beta-blockers, corticosteroids, and iodine compounds is recommended as standard practice [11].

## Conclusions

TSs represent a rare but critical endocrine emergency that may be precipitated by a variety of physiological stressors, including traumatic injuries. The presented case underscores the necessity for clinicians to maintain a high index of suspicion for TS in trauma patients, particularly those exhibiting unexplained hemodynamic instability in the absence of a known thyroid disorder. Timely identification and initiation of appropriate, targeted therapeutic interventions are essential to minimize associated morbidity and mortality. When clinical deterioration cannot be fully accounted for by traumatic injuries alone, TS should be considered as a potential differential diagnosis, as early recognition can be pivotal in improving patient outcomes.
